# The environment dependent dilaton in the laboratory and the solar system

**DOI:** 10.1140/epjc/s10052-022-10905-w

**Published:** 2022-10-20

**Authors:** Philippe Brax, Hauke Fischer, Christian Käding, Mario Pitschmann

**Affiliations:** 1 Institut de Physique Théorique, Université Paris-Saclay, CEA, CNRS, 91191 Gif/Yvette Cedex, France; 2 Atominstitut, Technische Universität Wien, Stadionallee 2, 1020 Wien, Austria

## Abstract

We consider the environment-dependent dilaton in the laboratory and the solar system and derive approximate analytical solutions to the field theory equations of motion in the presence of a one or two mirror system or a sphere. The solutions obtained herein can be applied to *q*BOUNCE experiments, neutron interferometry and for the calculation of the dilaton field induced “Casimir force” in the Cannex experiment as well as for Lunar Laser Ranging. They are typical of the Damour–Polyakov screening mechanism whereby deviations from General Relativity are suppressed by a vanishingly small direct coupling of the dilaton to matter in dense environments. We specifically focus on dilaton models which are compatible with the late time acceleration of the expansion of the Universe, i.e. the cosmological dilaton. We show how future laboratory experiments will essentially test a region of parameter space with $A_2\simeq \lambda ^2$ where $A_2$ is the quadratic coupling strength of the dilaton to matter and $\lambda$ is the steepness of the exponential runaway potential. Current constraints favour the large $A_2$ regime implying that the environment-dependent dilaton satisfies two of the swampland conjectures, i.e. the distance conjecture whereby the field excursion should not exceed the Planck scale and the de Sitter conjecture specifying that the running dilaton potential should be steep enough with a large $\lambda$.

## Introduction

The accelerated expansion of the Universe may require the introduction of additional degrees of freedom (see [1] for a recent review). Such new degrees of freedom, in particular light scalars, are theoretically well motivated irrespective of their role for the acceleration of the expansion of the Universe. If they exist in Nature, they must either only be feebly coupling to other matter or appear in some screened form in order to prevent detection in all past experiments and observations involving scalar fifth forces. A number of screening mechanisms exist [1], the chameleon [2–4] and Damour–Polyakov mechanisms [5], the K-mouflage [6–8] and Vainshtein ones [9], allowing such hypothetical fields to remain unseen in local tests of gravity.

In the companion paper [10], gravity resonance spectroscopy [11, 12], a Casimir force experiment [13] and Lunar Laser Ranging (LLR) [14] are used for the first time to put new bounds on the environment-dependent dilaton [15, 16]. The LLR bounds have already been discussed for chameleon [17] and symmetron scalar fields [18]. The dilaton model conjugates two ingredients. First of all, it involves an exponentially decreasing potential as expected in the strong coupling limit of string theory [19–21] and second it is the simplest realisation of the least coupling principle as advocated in [5]. Hence this model is a prime example, with the symmetron, of a model subject to the Damour–Polyakov screening mechanism [22]. The experimental analysis in [10] depends heavily on the field profile of dilatons in both the one mirror or two mirror setups, where the field is either present over an infinite plane of high density or is confined between two such parallel planes. One of the purposes of this paper is to provide a detailed analysis of the environment-dependent dilaton in realistic situations which can be tested using current experiments. For instance the analysis of the LLR results as well as the screening of neutrons in Q-bounce experiments employ the dilaton solution around and inside a sphere as derived here. For a similar study applied to chameleon field theories see [23]. The symmetron field equations have been studied in a similar fashion in [24, 25]. Testing screened models can be tackled from the laboratory to cosmological scales as reviewed in [26]. The parameter space of the environment-dependent dilaton has already been partially analysed in [15] where large values of $A_2$, the quadratic coupling to matter, and $\lambda$, the steepness of the dilaton runaway potential, were favoured. In this paper, we provide the tools to analyse the parameter space of the environment-dependent dilaton in different contexts, in particular in laboratory experiments. As we shall see, the different experiments probe different length scales and therefore can test the dilatons with related mass scales. This is already what happens for the symmetrons [27].

The environment dependent dilaton is inspired by string theory in the strong coupling regime and therefore should satisfy the swampland conjectures [28]. This issue was considered in [29] from the cosmological point of view. Here we analyse the excursion of the dilaton in the laboratory and the solar system. We confirm that the field covers sub-Planckian distances. Moreover, the region of parameter space where the dilaton has an impact in the laboratory and the solar system is such that the de Sitter conjecture is satisfied. This guarantees that the dilaton runaway potential is steep enough. As a result, the experimental tests of the environment-dependent dilaton [10] will probe a theory which is not in the swampland.

In Sect. 2 we will provide some background information on dilatons, which will provide the relevant definitions for the field theory analysis. We consider a two-dimensional parameter subspace of the dilaton model, which is of special cosmological significance as noted in Sect. 3. Then, in Sect. 4 approximate solutions for the one mirror case will be derived, while in Sect. 5 the corresponding two mirror solutions will be given. In Sect. 6 approximate dilaton solutions are derived for a spherical source. Section 7 provides relevant information on the *q*BOUNCE experiment, where the dilaton-induced resonance frequency shift for the case of a single mirror has also been summarized for a large range of parameters. In Sect. 8 the induced pressure in Casimir experiments due to the dilaton field between two mirrors of the experimental setup is derived, while in Sect. 9 bounds due to LLR are given. A conclusion in Sect. 10 will be followed by Appendix A, in which the precession of the lunar perigee induced by fifth forces is obtained.

## Background

Following [30], the dilaton potential is given by

1 $$\begin{aligned} V(\phi ) = V_0\,e^{-\lambda \phi /m_\text {Pl}}\>, \end{aligned}$$

where $V_0$ is an energy scale related to the dark energy of the Universe and $\lambda$ a numerical constant. This potential corresponds to the string theory dilaton potential in the strong coupling limit [5, 20]. Together with the coupling to matter this induces an effective potential

2 $$\begin{aligned} V_\text {eff}(\phi ; \rho ) = V(\phi ) + A(\phi )\,\rho \>, \end{aligned}$$

where for the environment-dependent dilaton we have [15]

3 $$\begin{aligned} A(\phi ) = 1 + \frac{A_2}{2m_\text {Pl}^2}\,\phi ^2\>, \end{aligned}$$

and hence

4 $$\begin{aligned} V_\text {eff}(\phi ; \rho ) = V_0\,e^{-\lambda \phi /m_\text {Pl}} + \frac{A_2\rho }{2m_\text {Pl}^2}\,\phi ^2\>. \end{aligned}$$

Here, we have neglected an additional term $\rho$, which does not affect the equations of motion. The minimum value $\phi _\rho$ in the presence of a density $\rho$ is given by $V_{\text {eff},\phi }(\phi ; \rho )\big |_{\phi =\phi _\rho }=0$ and reads

5 $$\begin{aligned} \phi _\rho = \frac{m_\text {Pl}}{\lambda }\,W\bigg (\frac{\lambda ^2V_0}{A_2\rho }\bigg ) \end{aligned}$$

with the Lambert *W*-function

6 $$\begin{aligned} W(x)&= \sum _{n=1}^\infty \frac{(-n)^{n-1}}{n!}\,x^n \nonumber \\&= x - x^2 + \frac{3}{2}\,x^3 - \frac{8}{3}\,x^4 + \frac{125}{24}\,x^5 - \frac{54}{5}\,x^6 + \cdots \>. \end{aligned}$$

For large arguments the approximative relation holds:

7 $$\begin{aligned} W(x) \simeq \ln x\>. \end{aligned}$$

The mass $\mu _\rho$ of the quantum fluctuation is therefore

8 $$\begin{aligned} \mu _\rho&= \sqrt{V_{\text {eff},\phi \phi }(\phi _\rho ; \rho )} \nonumber \\&=\frac{1}{m_\text {Pl}}\sqrt{\lambda ^2V_0\,e^{-\lambda \phi _\rho /m_\text {Pl}} + A_2\rho }\>. \end{aligned}$$

We employ the metric signature ($+---$), for which the stress-energy tensor of a scalar field is

9 $$\begin{aligned} T_{\mu \nu }^\phi&= \partial _\mu \phi \,\partial _\nu \phi - g_{\mu \nu }\Big (\frac{1}{2}\,\partial _\alpha \phi \,\partial ^\alpha \phi - V(\phi ; \rho )\Big )\>, \end{aligned}$$

while the equations of motion read

10 $$\begin{aligned} \Box \phi + V_{\text {eff},\phi }(\phi ; \rho ) = 0\> \end{aligned}$$

in the presence of matter.

In the following, we will require that $\lambda > rsim 1$ to satisfy the de Sitter conjecture and check that the field excursion $\Delta \phi$ is always smaller than the Planck scale as requested by the swampland conjecture. We will find that these two requirements are satisfied in the interesting part of the dilaton parameter space where the models can be tested. In a nutshell, the distance conjecture will follow from the influence of the coupling to matter which forces the field to remain close to the origin in field space when matter is present.

## The “Cosmological” dilaton

The parameter space of the dilaton model is 3-dimensional ($V_0$, $\lambda$, $A_2$). Since we are interested only in “cosmological” dilatons, having significance in the cosmological domain, we consider only the 2-dimensional parameter subspace, for which

11 $$\begin{aligned} V_\text {eff}(\phi _V; \rho _V) = 3\Omega _{\Lambda 0}m_\text {Pl}^2H_0^2 = 6.73\times 10^{-34}\>\text {MeV}^4\>, \end{aligned}$$

where $\phi _V$, $\rho _V$ are the corresponding vacuum values and the density parameter $\Omega _{\Lambda 0} \sim 0.73$. This choice neglects the possible instability of the potential under radiative corrections. We take the parameter $V_0$ as a function of $A_2$ and $\lambda$ such that Eq. (11) is obeyed. It is important to notice that the coupling function involves the strong coupling scale

12 $$\begin{aligned} M= \frac{m_{\textrm{Pl}}}{\sqrt{A}_2}. \end{aligned}$$

In the spirit of an effective field theory expansion of the coupling function, one must require that $\phi /M \ll 1$ to guarantee that terms of higher order in $\phi$ can be neglected. Similarly the exponential potential involves the scale

13 $$\begin{aligned} \Lambda = \frac{m_{\textrm{Pl}}}{\lambda } \end{aligned}$$

and one must make sure that typically $\phi /\Lambda \gg 1$ to guarantee that higher order string corrections in $e^{-n \phi /\Lambda }$ can be neglected. We will make sure that these conditions are satisfied in the following. As long as $A_2 > rsim 1$ and $\lambda > rsim 1$, the previous conditions guarantee that the distance and de Sitter conjectures are valid.

It is straightforward to show that the condition (11) leads to

14 $$\begin{aligned} V_0(A_2, \lambda )&= \frac{A_2\rho _V}{\lambda ^2}\left( \sqrt{1 + \frac{2\lambda ^2}{A_2\rho _V}\,3\Omega _{\Lambda 0}m_\text {Pl}^2H_0^2} - 1\right) \nonumber \\&\quad \times \exp \left\{ \sqrt{1 + \frac{2\lambda ^2}{A_2\rho _V}\,3\Omega _{\Lambda 0}m_\text {Pl}^2H_0^2} - 1\right\} \>. \end{aligned}$$

**Fig. 1:**
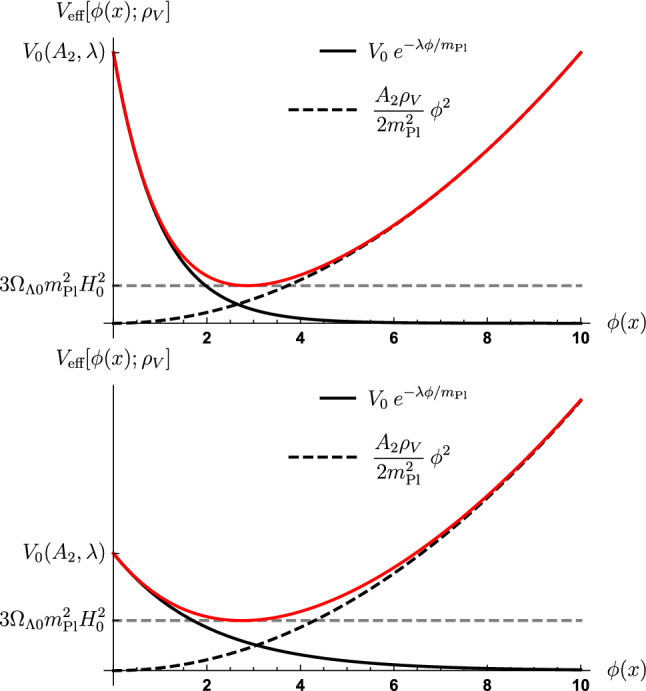
The effective potential in Eq. (4) is plotted with its components for two different sets of parameter values for $\lambda$ and $A_2$, while the parameter $V_0$ varies as a function of $A_2$ and $\lambda$ such that the effective potential of the minimum in vacuum equals $3\Omega _{\Lambda 0}m_\text {Pl}^2H_0^2$

The dilaton parameter space becomes effectively 2-dimensional ($\lambda$, $A_2$) simplifying also the representation of experimental constraints. For illustrative purposes, in Fig. 1 the effective potential with its components is depicted for two different sets of parameter values for $\lambda$ and $A_2$.

Using Eq. (14) in Eq. (8) the mass $\mu _\rho$ may be expressed as a function of the parameters $\lambda$ and $A_2$ as well

15 $$\begin{aligned} \mu _\rho&= \frac{\sqrt{A_2\rho }}{m_\text {Pl}}\,\left\{ 1 + W\left[ \frac{\rho _V}{\rho }\left( \sqrt{1 + \frac{2\lambda ^2}{A_2\rho _V}\,3\Omega _{\Lambda 0}m_\text {Pl}^2H_0^2} - 1\right) \right. \right. \nonumber \\&\quad \times \left. \left. \exp \left( \sqrt{1 + \frac{2\lambda ^2}{A_2\rho _V}\,3\Omega _{\Lambda 0}m_\text {Pl}^2H_0^2} - 1\right) \right] \right\} ^{1/2}\>. \end{aligned}$$

With these analytical expressions we can perform the limits $A_2\rightarrow \infty$, $\lambda \rightarrow \infty$, $A_2\rightarrow 0$ or $\lambda \rightarrow 0$ and study the behavior along curves $\lambda \propto \sqrt{A_2}$, which all provide some qualitative insight of the relevant parameter region. This behavior is summarized in the following two Tables 1 and 2.

**Table 1:** Here, Eqs. (5) and (8) are given for different limits

	$\phi _\rho$	$\mu _\rho$
$\lim _{A_2\rightarrow \infty }$	0	$\infty$
$\lim _{\lambda \rightarrow \infty }$	$\displaystyle m_\text {Pl}\,\sqrt{\frac{2}{A_2\rho _V}\,3\Omega _{\Lambda 0}m_\text {Pl}^2H_0^2}$	$\infty$
$\lim _{A_2\rightarrow 0}$	$\infty$	0
$\lim _{\lambda \rightarrow 0}$	0	$\displaystyle \frac{\sqrt{A_2\rho }}{m_\text {Pl}}$
$\lambda \propto \sqrt{A_2}$	$\sim 1/\lambda$	$\sim \lambda$

**Table 2:** The limits of the two constituent parts of the effective potential Eq. (4) are summarized in this table

	$\displaystyle V_0\,e^{-\lambda \phi _\rho /m_\text {Pl}}$	$\displaystyle \frac{A_2\rho }{2m_\text {Pl}^2}\,\phi _\rho ^2$
$\lim _{A_2\rightarrow \infty }$	$3\Omega _{\Lambda 0}m_\text {Pl}^2H_0^2$	0
$\lim _{\lambda \rightarrow \infty }$	0	$\displaystyle \frac{\rho }{\rho _V}\,3\Omega _{\Lambda 0}m_\text {Pl}^2H_0^2$
$\lim _{A_2\rightarrow 0}$	0	$\displaystyle \frac{\rho }{\rho _V}\,3\Omega _{\Lambda 0}m_\text {Pl}^2H_0^2$
$\lim _{\lambda \rightarrow 0}$	$3\Omega _{\Lambda 0}m_\text {Pl}^2H_0^2$	0
$\lambda \propto \sqrt{A_2}$	const	const

We may summarize the findings of the tables as follows. For roughly $\lambda \propto \sqrt{A_2}$ we expect finite limits to be obtainable from experiments as long as $\lambda$ is not too large since otherwise $\phi _\rho$ decreases, i.e. the dilaton effectively disappears and the interaction range $1/\mu _\rho$ vanishes as well. For $A_2 \gg \lambda$ we expect no limits from experiments since $\phi _\rho$ becomes small. On the other hand, for $\lambda \gg A_2$ we also do not expect bounds since in this case either $1/\mu _\rho$ decreases without limit or $\phi _\rho$ diverges and cannot act dynamically anymore. Hence, we find that the dilaton has a physical impact only within a restricted region in the $A_2$, $\lambda$ parameter space. Consequently, all experimental constraints have to lie within this region. As can be seen in Table 1, shorter interaction ranges correspond to larger values of $A_2$ and $\lambda$. Therefore, we expect LLR to probe small parameter values within this region, table top experiments correspondingly larger parameters and collider constraints still larger values. Numerical evaluations in the accompanying article [10] corroborate these findings. Notice that when $\lambda = \kappa \sqrt{A}_2$, the validity of the model as an effective field theory is guaranteed when $\kappa \ll 1$ as the field values must satisfy $\Lambda \ll \phi \ll M$ where $\Lambda = \kappa M \ll M$. These expectations are also confirmed by the actual bounds in [10].

## One mirror

In this section, we treat the case of a single mirror filling the infinite half-space $z \le 0$. The 1-dimensional equation of motion reads (see Eq. (10))

16 $$\begin{aligned} \frac{d^2\phi }{dz^2} = V_{\text {eff},\phi }(\phi ; \rho )\>. \end{aligned}$$

Multiplication by $\phi '$ and integration with respect to *z* gives

17 $$\begin{aligned} \frac{1}{2}\left( \frac{d\phi }{dz}\right) ^2 - \frac{1}{2}\left( \frac{d\phi }{dz}\right) ^2\bigg |_{z=z_0} = V_\text {eff}(\phi ; \rho ) - V_\text {eff}(\phi ; \rho )\big |_{z=z_0}\>, \end{aligned}$$

with the integration constant $z_0$ and leads to

18 $$\begin{aligned}{} & {} \int _{\phi _0}^{\phi (z)}\frac{d\phi }{\displaystyle \sqrt{V_\text {eff}(\phi ; \rho ) - V_\text {eff}(\phi ; \rho )\big |_{z=z_0} + \frac{1}{2}\left( \frac{d\phi }{dz}\right) ^2\bigg |_{z=z_0}}} \nonumber \\{} & {} \quad = \pm \sqrt{2}\left( z - z_0\right) , \end{aligned}$$

where $\phi _0=\phi (z_0)$, the $+$ sign is for $\phi ' \ge 0$, while the − sign holds for $\phi ' \le 0$.

### The vacuum region

First, we consider the case of low density $\rho _V$ corresponding to the medium above the mirror and search for a solution that asymptotically for $z \rightarrow \infty$ goes as $\phi (z) \rightarrow \phi _V$ with

19 $$\begin{aligned} \phi _V = \frac{m_\text {Pl}}{\lambda }\,W\bigg (\frac{\lambda ^2V_0}{A_2\rho _V}\bigg )\>, \end{aligned}$$

implying $\phi ' \rightarrow 0$. We find for $z_0=0$ and $z\rightarrow \infty$ from Eq. (17)

20 $$\begin{aligned} - \frac{1}{2}\left( \frac{d\phi }{dz}\right) ^2\bigg |_{z=0} = V_\text {eff}(\phi _V; \rho _V) - V_\text {eff}(\phi ; \rho _V)\big |_{z=0}\>. \end{aligned}$$

Using Eq. (20) in Eq. (18) gives

21 $$\begin{aligned} \int _{\phi _0}^{\phi (z)}\frac{d\phi }{\displaystyle \sqrt{V_\text {eff}(\phi ; \rho _V) - V_\text {eff}(\phi _V; \rho _V)}} = \sqrt{2}\,z\>, \end{aligned}$$

respectively

22 $$\begin{aligned}&\int _{\phi _0}^{\phi (z)}\frac{d\phi }{\sqrt{\displaystyle V_0\left( e^{-\lambda \phi /m_\text {Pl}} - e^{-\lambda \phi _V/m_\text {Pl}}\right) + \frac{A_2\rho _V}{2m_\text {Pl}^2}\left( \phi ^2 - \phi _V^2\right) }} \nonumber \\&\quad = \sqrt{2}\,z\>. \end{aligned}$$

Approximating the effective potential around its minimum at the vacuum value $\phi _V$, we find to leading order

23 $$\begin{aligned} \frac{1}{\mu _V}\int _{(\phi _V - \phi (z))/m_\text {Pl}}^{(\phi _V - \phi _0)/m_\text {Pl}}\frac{d{{\tilde{\phi }}}}{{{\tilde{\phi }}}} = z \end{aligned}$$

with the mass of the quantum fluctuation in vacuum

24 $$\begin{aligned} \mu _V&= \sqrt{V_{\text {eff},\phi \phi }(\phi ; \rho _V)}\Big |_{\phi =\phi _V} \nonumber \\&=\frac{1}{m_\text {Pl}}\sqrt{\lambda ^2V_0\,e^{-\lambda \phi _V/m_\text {Pl}} + A_2\rho _V}\>. \end{aligned}$$

Inverting the relation straightforwardly leads to

25 $$\begin{aligned} \phi (z) = \phi _V + \left( \phi _0 - \phi _V\right) e^{-\mu _Vz}\>. \end{aligned}$$

### The high density region

Here, we consider the case of high density $\rho _M$ as inside the mirror. Clearly, for $z\rightarrow -\infty$ we have $\phi (z)\rightarrow \phi _M$ with

26 $$\begin{aligned} \phi _M = \frac{m_\text {Pl}}{\lambda }\,W\bigg (\frac{\lambda ^2V_0}{A_2\rho _M}\bigg )\>, \end{aligned}$$

and hence $\phi '\rightarrow 0$. Therefore, we find for $z_0=0$ and $z\rightarrow -\infty$ from Eq. (17)

27 $$\begin{aligned} - \frac{1}{2}\left( \frac{d\phi }{dz}\right) ^2\bigg |_{z=0} = V_\text {eff}(\phi _M; \rho _M) - V_\text {eff}(\phi ; \rho _M)\big |_{z=0}\>. \end{aligned}$$

Using Eq. (27) in Eq. (18) gives

28 $$\begin{aligned} \int _{\phi _0}^{\phi (z)}\frac{d\phi }{\displaystyle \sqrt{V_\text {eff}(\phi ; \rho _M) - V_\text {eff}(\phi _M; \rho _M)}} = \sqrt{2}\,z\>. \end{aligned}$$

Proceeding analogously to the case of vacuum, we finally obtain

29 $$\begin{aligned} \phi (z) = \phi _M + \left( \phi _0 - \phi _M\right) e^{\mu _Mz}\>, \end{aligned}$$

with the mass of the quantum fluctuation in the mirror

30 $$\begin{aligned} \mu _M = \frac{1}{m_\text {Pl}}\sqrt{\lambda ^2V_0\,e^{-\lambda \phi _M/m_\text {Pl}} + A_2\rho _M}\>. \end{aligned}$$

### Boundary conditions

Using the boundary conditions

31 $$\begin{aligned} \frac{d\phi }{dz}\bigg |_{z=0_-} = \frac{d\phi }{dz}\bigg |_{z=0_+}\>, \end{aligned}$$

we find

32 $$\begin{aligned} \phi _0 = \frac{\mu _V\,\phi _V + \mu _M\,\phi _M}{\mu _V + \mu _M}\>. \end{aligned}$$

The second boundary condition

33 $$\begin{aligned} \phi (0_-) = \phi (0_+)\>, \end{aligned}$$

is trivially satisfied.

### Final solution

Summarising, we obtain the solution

34 $$\begin{aligned} \phi (z)&= \Theta (+z)\,\big (\phi _V + \left( \phi _0 - \phi _V\right) e^{-\mu _Vz}\big ) \nonumber \\&\quad + \Theta (-z)\,\big (\phi _M + \left( \phi _0 - \phi _M\right) e^{\mu _Mz}\big )\>, \end{aligned}$$

with

35 $$\begin{aligned} \mu _V&=\frac{1}{m_\text {Pl}}\sqrt{\lambda ^2V_0\,e^{-\lambda \phi _V/m_\text {Pl}} + A_2\rho _V}\>, \nonumber \\ \mu _M&= \frac{1}{m_\text {Pl}}\sqrt{\lambda ^2V_0\,e^{-\lambda \phi _M/m_\text {Pl}} + A_2\rho _M}\>, \end{aligned}$$

and

36 $$\begin{aligned} \phi _0 = \frac{\mu _V\,\phi _V + \mu _M\,\phi _M}{\mu _V + \mu _M}\>. \end{aligned}$$

**Fig. 2:**
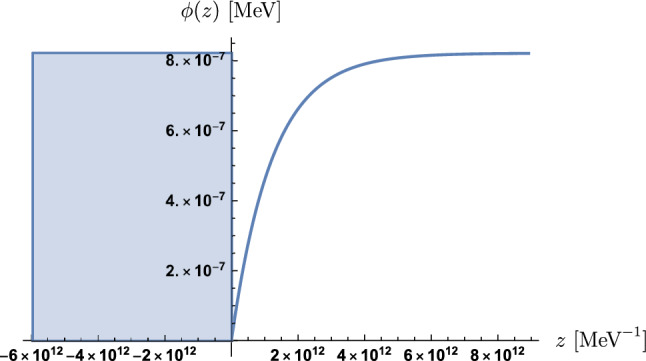
This plot shows the dilaton solution with the blue area depicting the mirror of the experimental setup. We can see that $\lambda \phi (z) \ll m_\text {Pl} = 1.22\times 10^{22}$ MeV is indeed satisfied in this case

A prototype solution with values $A_2 = 10^{35}$, $\lambda = 10^{25}$ and $V_0 = 1.00034\times 3\Omega _{\Lambda 0}m_\text {Pl}^2H_0^2 = 6.73\times 10^{-10}$ meV$^4$, $\rho _V = 10^{-15}$ MeV$^4$ and $\rho _M = 1.082\times 10^{-5}$ MeV$^4$ is plotted in Fig. 2.

As already argued, we see in Fig. 2 that the field excursion is sub-Planckian. Moreover, these dilaton solutions are instrumental in analyzing the results of *q*BOUNCE experiments as outlined in Sect. 7.

## Two mirrors

In this section, we treat the case of two parallel infinitely thick mirrors separated at distance 2*d* in *z*-direction with $z=0$ being the center between the two mirrors.

### The vacuum region

Here, we consider the case of low density $\rho _V$, as between the mirrors and choose $z_0=0$. Due to the symmetry of the setup, the derivative of the field has to vanish there yielding for Eq. (18)

37 $$\begin{aligned} \int _{\phi _0}^{\phi (z)}\frac{d\phi }{\displaystyle \sqrt{V_\text {eff}(\phi ; \rho _V) - V_\text {eff}(\phi _0; \rho _V)}} = \sqrt{2}\,z\>, \end{aligned}$$

where $\phi _0 = \phi (0)$. Approximating the potential around $\phi _0$ yields

38 $$\begin{aligned}&V_\text {eff}(\phi ; \rho _V) - V_\text {eff}(\phi _0; \rho _V) \nonumber \\&\quad \simeq -{\mathfrak {D}}_0\left( \phi - \phi _0\right) + \frac{\mu _0^2}{2}\left( \phi - \phi _0\right) ^2\>, \end{aligned}$$

with the positive constants

39 $$\begin{aligned} {\mathfrak {D}}_0&= \frac{\lambda V_0}{m_\text {Pl}}\,e^{-\lambda \phi _0/m_\text {Pl}} - \frac{A_2\rho _V}{m_\text {Pl}^2}\,\phi _0\>, \nonumber \\ \mu _0&= \frac{1}{m_\text {Pl}}\sqrt{\lambda ^2V_0\,e^{-\lambda \phi _0/m_\text {Pl}} + A_2\rho _V}\>, \end{aligned}$$

leads to

40 $$\begin{aligned} \int _{\phi _0}^{\phi (z)}\frac{d\phi }{\sqrt{\displaystyle -{\mathfrak {D}}_0\left( \phi - \phi _0\right) + \frac{\mu _0^2}{2}\left( \phi - \phi _0\right) ^2}} = -\sqrt{2}\,|z|\>. \end{aligned}$$

Substituting $\displaystyle x = 1 + \frac{\mu _0^2}{{\mathfrak {D}}_0}\left( \phi _0 - \phi \right)$ leads to the elementary integral

41 $$\begin{aligned} \frac{1}{\mu _0}\int _{1}^{1 + \frac{\mu _0^2}{{\mathfrak {D}}_0}\,(\phi _0 - \phi (z))}\frac{dx}{\sqrt{\displaystyle x^2 - 1}} = |z|\>. \end{aligned}$$

Integration and some transformations lead to the solution

42 $$\begin{aligned} \phi (z) = \phi _0 + \frac{{\mathfrak {D}}_0}{\mu _0^2}\,\big (1 - \cosh (\mu _0z)\big )\>. \end{aligned}$$

### The high density region

We can read off the solution inside the mirrors directly from the corresponding solution in the one mirror case Eq. (34)

43 $$\begin{aligned} \phi (z)&= \phi _M + \left( \phi _d - \phi _M\right) e^{-\mu _M(|z| - d)}\>, \end{aligned}$$

where $\phi _d = \phi (d) = \phi (-d)$ is the field value at the surfaces of the mirrors.

### Boundary conditions

Using the boundary conditions at the mirror surface

44 $$\begin{aligned} \phi (d_-) = \phi (d_+)\>, \end{aligned}$$

gives $\phi _d$ as a function of $\phi _0$

45 $$\begin{aligned} \phi _d = \phi _0 + \frac{{\mathfrak {D}}_0}{\mu _0^2}\left( 1 - \cosh (\mu _0d)\right) \>, \end{aligned}$$

which together with the second boundary condition

46 $$\begin{aligned} \frac{d\phi }{dz}\bigg |_{z=d_-} = \frac{d\phi }{dz}\bigg |_{z=d_+} \end{aligned}$$

gives an equation, which defines $\phi _0$ implicitly

47 $$\begin{aligned} \phi _M + \frac{{\mathfrak {D}}_0}{\mu _0\mu _M}\sinh (\mu _0d) = \phi _0 + \frac{{\mathfrak {D}}_0}{\mu _0^2}\left( 1 - \cosh (\mu _0d)\right) \end{aligned}$$

since ${\mathfrak {D}}_0$ and $\mu _0$ both are functions of $\phi _0$.

### Final solution

Summarising, we obtain the solution

48 $$\begin{aligned} \phi (z)&= \Theta (d - |z|)\left\{ \phi _0 + \frac{{\mathfrak {D}}_0}{\mu _0^2}\,\big (1 - \cosh (\mu _0z)\big )\right\} \nonumber \\&\quad + \Theta (|z| - d)\left\{ \phi _M + \left( \phi _d - \phi _M\right) e^{-\mu _M(|z| - d)}\right\} \>, \end{aligned}$$

where

49 $$\begin{aligned} \mu _M&= \frac{1}{m_\text {Pl}}\sqrt{\lambda ^2V_0\,e^{-\lambda \phi _M/m_\text {Pl}} + A_2\rho _M}\>, \nonumber \\ \mu _0&= \frac{1}{m_\text {Pl}}\sqrt{\lambda ^2V_0\,e^{-\lambda \phi _0/m_\text {Pl}} + A_2\rho _V}\>, \nonumber \\ {\mathfrak {D}}_0&= \frac{\lambda V_0}{m_\text {Pl}}\,e^{-\lambda \phi _0/m_\text {Pl}} - \frac{A_2\rho _V}{m_\text {Pl}^2}\,\phi _0\>, \end{aligned}$$

$\phi _d$ as a function of $\phi _0$ is given by

50 $$\begin{aligned} \phi _d = \phi _0 + \frac{{\mathfrak {D}}_0}{\mu _0^2}\left( 1 - \cosh (\mu _0d)\right) \>, \end{aligned}$$

and $\phi _0$ is solution to

51 $$\begin{aligned} \phi _M + \frac{{\mathfrak {D}}_0}{\mu _0\mu _M}\sinh (\mu _0d) = \phi _0 + \frac{{\mathfrak {D}}_0}{\mu _0^2}\left( 1 - \cosh (\mu _0d)\right) \>. \end{aligned}$$

**Fig. 3:**
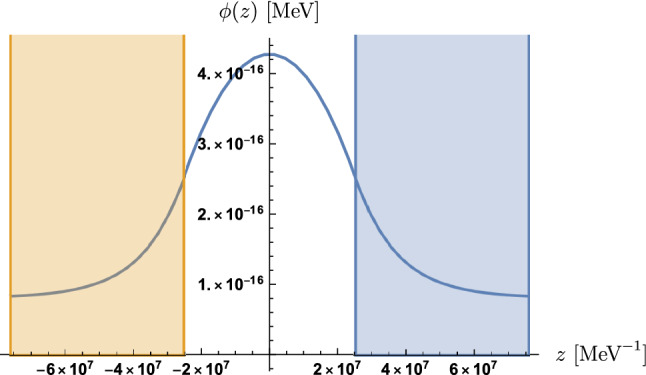
This plot shows the dilaton solution with the blue and orange area depicting the mirrors of the experimental setup. We can see that $\lambda \phi (z) \ll m_\text {Pl} = 1.22\times 10^{22}$ MeV is indeed satisfied in this case

A prototype solution is plotted in Fig. 3. The induced pressure inside the planes is $\displaystyle P = -4.73\times 10^{-4}$ pN/cm$^2$ (see Sect. 7).

Once again we see in Fig. 3 that the field excursion is sub-Planckian. These dilaton solutions will be use to evaluate the pressure in Casimir experiments.

## Dilaton field of a sphere

In this section we consider the dilaton field as caused by a static massive sphere surrounded by vacuum with radius *R* and a homogenous high density $\rho _S$, which is used for the analysis of the *q*BOUNCE and Lunar Laser Ranging bounds.

Since neutrons are used in the search for dilatons in *q*BOUNCE experiments and neutron interferometry, it is important to understand their interaction with the dilaton. For a certain parameter regime this interaction between neutron and dilaton becomes strong. In this case, the neutron affects the background dilaton field as generated by the mirrors of the experimental setup in a non-negligible way, which in turn weakens the effect on the neutron, *viz.* screening of the neutron sets in (see also [31]). Since we treat the dilaton as a classical field theory, a consistent description of its coupling to a quantum mechanical system is beyond our reach. Therefore, we employ a semi-classical treatment in which the neutron’s probability distribution times its mass acts as the source of the dilaton as defined in Eq. (81).

Concerning Lunar Laser Ranging, we approximate the Sun, Earth and Moon as spherical sources of dilatons with homogenous density and employ the solutions derived in this section.

The spherically symmetric field equation is given by

52 $$\begin{aligned} \frac{d^2\phi }{dr^2} + \frac{2}{r}\frac{d\phi }{dr} = V_{\text {eff},\phi }(\phi ; \rho )\>, \end{aligned}$$

with the boundary conditions

53 $$\begin{aligned} \phi '(0)&= 0\>, \nonumber \\ \lim _{r\rightarrow \infty }\phi (r)&\rightarrow \phi _V\>. \end{aligned}$$

Inside the sphere we have density $\rho _S$. The corresponding minimum of the field value is

54 $$\begin{aligned} \phi _S = \frac{m_\text {Pl}}{\lambda }\,W\bigg (\frac{\lambda ^2V_0}{A_2\rho _S}\bigg )\>. \end{aligned}$$

We expand

55 $$\begin{aligned} V_{\text {eff},\phi }(\phi ; \rho _S)&\simeq V_{\text {eff},\phi \phi }(\phi _S; \rho _S)\left( \phi - \phi _S\right) \nonumber \\&\quad = \mu _S^2\left( \phi - \phi _S\right) \end{aligned}$$

with

56 $$\begin{aligned} \mu _S = \frac{1}{m_\text {Pl}}\sqrt{\lambda ^2 V_0\,e^{-\lambda \phi _S/m_\text {Pl}} + A_2\rho _S}\>. \end{aligned}$$

For the field equation we find

57 $$\begin{aligned} \frac{d^2\phi }{dr^2} + \frac{2}{r}\frac{d\phi }{dr} \simeq \mu _S^2\left( \phi - \phi _S\right) . \end{aligned}$$

Introducing the field $\varphi$

58 $$\begin{aligned} \phi - \phi _S = \frac{\varphi }{r}\>, \end{aligned}$$

Eq. (57) takes on the simpler form

59 $$\begin{aligned} \frac{d^2\varphi }{dr^2} \simeq \mu _S^2\,\varphi \>. \end{aligned}$$

From the general solution for $\varphi$ we obtain the particular solution for $\phi$, which is convergent for $r\rightarrow 0$ and satisfies the boundary condition $\phi '(0) = 0$:

60 $$\begin{aligned} \phi (r) = \phi _S + {\mathfrak {C}}_I\frac{\displaystyle \sinh (\mu _Sr)}{r}\>, \end{aligned}$$

where ${\mathfrak {C}}_I$ is some constant to be determined later.

In the vacuum outside the sphere we approximate

61 $$\begin{aligned} V_{\text {eff},\phi }(\phi ; \rho _V)&\simeq V_{\text {eff},\phi \phi }(\phi _V; \rho _V)\left( \phi - \phi _V\right) \nonumber \\&= \mu _V^2\left( \phi - \phi _V\right) , \end{aligned}$$

where $\mu _V$ is given by Eq. (24).

For the field equation we find

62 $$\begin{aligned} \frac{d^2\phi }{dr^2} + \frac{2}{r}\frac{d\phi }{dr} \simeq \mu _V^2\left( \phi - \phi _V\right) . \end{aligned}$$

With the field $\varphi$

63 $$\begin{aligned} \phi - \phi _V = \frac{\varphi }{r}\>, \end{aligned}$$

the field equation again simplifies to

64 $$\begin{aligned} \frac{d^2\varphi }{dr^2} \simeq \mu _V^2\,\varphi \>. \end{aligned}$$

The general solution of which provides the $\phi$ solution convergent for $r\rightarrow \infty$ and satisfying the boundary condition $\lim _{r\rightarrow \infty }\phi (r) \rightarrow \phi _V$

65 $$\begin{aligned} \phi (r) = \phi _V + {\mathfrak {C}}_O\frac{e^{-\mu _V (r - R)}}{r}\>, \end{aligned}$$

where ${\mathfrak {C}}_O$ is some constant to be determined next.

The dilaton field has to satisfy the following boundary conditions at the surface of the sphere

66 $$\begin{aligned} \phi (R_-)&= \phi (R_+)\>, \nonumber \\ \phi '(R_-)&= \phi '(R_+)\>, \end{aligned}$$

which provides explicit expressions for ${\mathfrak {C}}_I$ and ${\mathfrak {C}}_O$. After some elementary algebraical manipulations we obtain the relations

67 $$\begin{aligned} {\mathfrak {C}}_I&= \frac{\phi _V - \phi _S}{\cosh (\mu _SR)}\frac{1 + \mu _VR}{\mu _S + \mu _V\tanh (\mu _SR)}\>, \nonumber \\ {\mathfrak {C}}_O&= -R\,\frac{1 - \frac{1}{\mu _SR}\tanh (\mu _SR)}{1 + \frac{\mu _V}{\mu _S}\tanh (\mu _SR)}\left( \phi _V - \phi _S\right) \>. \end{aligned}$$

In order to quantify the amount of “screening” of a sphere, we introduce a formfactor, which we interpret as a “screening charge” ${\mathfrak {Q}}$. The interaction of the sphere with its surrounding is given by its outer field. Clearly, with decreasing radius *R* the sphere becomes increasingly “unscreened” since the dilaton field inside the sphere decreases towards its minimum throughout the volume of the sphere. On the other hand, for large sphere radii the dilaton field reaches its minimum already before the center of the sphere, it is no longer “sourced” by the whole volume of the sphere, which becomes “screened”.

An unscreened sphere has $R \ll 1/\mu _S$. Hence, we expand ${\mathfrak {C}}_O$ in powers of $\mu _SR$ to obtain for an “unscreened” sphere

68 $$\begin{aligned} {\mathfrak {C}}_O^u = -\frac{\mu _S^2R^3}{3}\left( \phi _V - \phi _S\right) \>. \end{aligned}$$

We define the “screening charge” ${\mathfrak {Q}}$ as the ratio

69 $$\begin{aligned} {\mathfrak {Q}} = \frac{{\mathfrak {C}}_O}{{\mathfrak {C}}_O^u} = \frac{3}{\mu _S^2R^2}\frac{1 - \frac{1}{\mu _SR}\tanh (\mu _SR)}{1 + \frac{\mu _V}{\mu _S}\tanh (\mu _SR)}\>. \end{aligned}$$

**Fig. 4:**
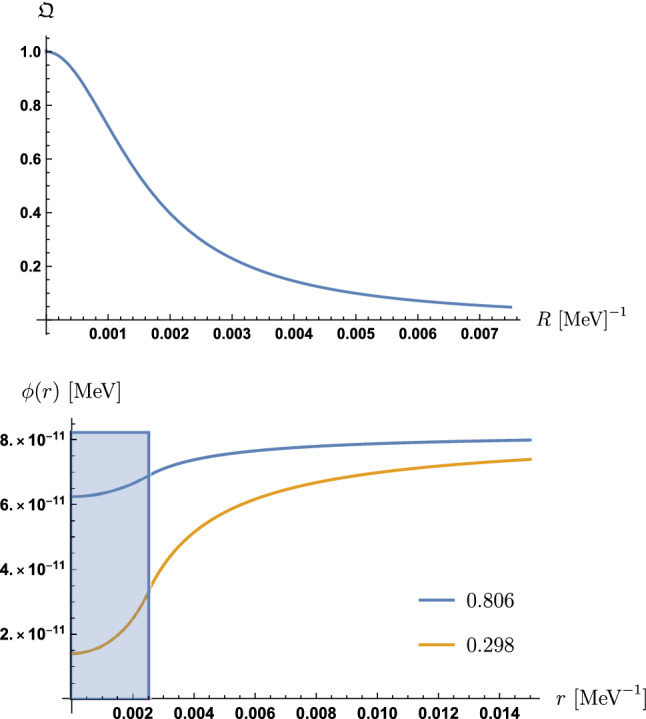
Top: The “screening charge” ${\mathfrak {Q}}$ is plotted as a function of the radius *R* of a sphere. The parameters taken are $\rho _N = 1.44\times 10^{10}$ MeV$^{4}$, the density of a neutron, $A_2 = 10^{40}$, $\lambda = 10^{15}$, $V_0$ according to Eq. (14) and *R* varying between 0 and $3\times R_N = 0.0075$ MeV$^{-1}$, where $R_N$ is the radius of a neutron. Bottom: The field profile of a sphere (neutron) is plotted as a function of the radial distance of the center of the sphere with parameters $A_2 = 10^{39}$, $\lambda = 10^{25}$ and $V_0$ according to Eq. (14). The blue line corresponds to the density of a neutron $\rho _N = 1.44\times 10^{10}$ MeV$^{4}$ providing a “screening charge” ${\mathfrak {Q}} = 0.806$ and the yellow line to $10\times \rho _N$ providing ${\mathfrak {Q}} = 0.298$. The blue square is bounded by the radius $R_N$ of the sphere (neutron) and the vacuum field value $\phi = \phi _V$

With this definition, see Fig. 4,

$$\begin{aligned} {\mathfrak {Q}} \rightarrow {\left\{ \begin{array}{ll} 0\>, &{} \quad \text {for ''screened'' bodies with } \mu _SR \rightarrow \infty \>,\\ 1\>, &{} \quad \text {for ''unscreened'' bodies with } \mu _SR \rightarrow 0\>. \end{array}\right. } \end{aligned}$$

Finally, we find for the solution

$$\begin{aligned} \phi (r) = {\left\{ \begin{array}{ll} &{}\phi _S + \frac{\phi _V - \phi _S}{\cosh (\mu _SR)}\frac{1 + \mu _VR}{\mu _S + \mu _V\tanh (\mu _SR)} \\ &{}\quad \quad \times \>\frac{\sinh (\mu _Sr)}{r}\>, \quad \quad \text {for } r\le R\>, \\ &{}\phi _V -{\mathfrak {Q}}\,\frac{\mu _S^2R^3}{3}\left( \phi _V - \phi _S\right) \frac{e^{-\mu _V(r - R)}}{r}\>, \text {for } r\ge R\>. \end{array}\right. } \end{aligned}$$

The acceleration experienced by a pointlike test body, which does not disturb the field, in the outer field of the sphere has been derived in [25] for a generic scalar $\phi$ in the non-relativistic limit as

70 $$\begin{aligned} \textbf{f}_\phi = -m\mathbf {\nabla }\ln A(\phi )\>. \end{aligned}$$

In all models of consideration $A(\phi ) = 1 + \delta A(\phi )$ with $\delta A(\phi )\ll 1$, such that $\ln A(\phi ) \simeq \delta A(\phi )$. Consequently, we find for the force on a particle caused by a scalar $\phi$ to leading order

71 $$\begin{aligned} \textbf{f}_\phi = -m\mathbf {\nabla }A(\phi )\>. \end{aligned}$$

In the case of dilatons Eq. (3) holds and we finally obtain for the dilaton force on a point particle

72 $$\begin{aligned} \textbf{f}_\phi =-m\,\frac{A_2}{m_\text {Pl}^2}\,\phi \,\mathbf {\nabla }\phi \>, \end{aligned}$$

and, respectively, for the acceleration

73 $$\begin{aligned} \textbf{a}_\phi = -\frac{A_2}{m_\text {Pl}^2}\,\phi \,\mathbf {\nabla }\phi \>. \end{aligned}$$

Asymptotically, for large *r* we obtain

74 $$\begin{aligned} \textbf{a}_\phi \rightarrow -{\mathfrak {Q}}\,\frac{A_2}{m_\text {Pl}^2}\frac{\mu _V\mu _S^2R^3}{3}\,\phi _V\left( \phi _V - \phi _S\right) \frac{e^{-\mu _V(r - R)}}{r}\frac{\textbf{r}}{r}\>, \end{aligned}$$

justifying the definition of ${\mathfrak {Q}}$ as a “screening charge”.

The screening effects make the field excursion sub-Planckian as seen in Fig. 4. The dilaton solutions will be used for the results about the *q*BOUNCE experiment and Lunar Laser Ranging in the following sections.

## Dilaton-induced frequency shift in *q*BOUNCE

Here, we derive a discrete set of limits for the *q*BOUNCE experiment [11, 12, 32] using the solutions obtained herein. In this experiment, ultra-cold neutrons are dropped in Earth’s gravitational potential and reflected by a neutron mirror, which has been reported for the first time in [33]. The energy eigenstates are discrete and allow to apply the method of resonance spectroscopy. In [12] the basic setup is described. In the Rabi version of the experiment, spectroscopy has been realized with energy resolution 3$\times$10$^{-15}$ peV [27].

The experimental setup is such, that ultracold neutrons pass three regions, while being reflected on polished glass mirrors. In [27], the resonance spectroscopy transitions between the energy ground state $E_1 = 1.41$ peV and the excited states $E_3 = 3.32$ peV as well as $E_4 = 4.08$ peV have been demonstrated. First, the neutrons pass region I which acts as a state selector for the ground state $|1\rangle$ having energy $E_1$. A polished mirror at the bottom and a rough absorbing scatterer on top at a height of about 20 $\mu$m serve to select the ground state. Neutrons in higher, unwanted states are scattered out of the system. This region has a length of 15 cm. Subsequently, in region II, a horizontal mirror performs harmonic oscillations with a tunable frequency $\omega$, which drives the system into a coherent superposition of ground and excited states. The length of this region is 20 cm. Finally, region III is identical to the first region and hence acts again as a ground state selector.

The quantum mechanical description of a neutron above a mirror in the gravitational potential is given by the Schrödinger equation [34]. After separation into free transversal and bound vertical states

75 $$\begin{aligned} \Psi _n^{(0)}({{\textbf {x}}},t) = \frac{e^{\frac{i}{\hbar }(p_\perp \cdot x_\perp - E_\perp t)}}{2\pi \hbar v_\perp }\,\psi _n^{(0)}(z)\,e^{-\frac{i}{\hbar }E_n t}\>, \end{aligned}$$

it reads

76 $$\begin{aligned} -\frac{\hbar ^2}{2m_N}\frac{\partial ^2\psi _n^{(0)}(z)}{\partial z^2} + m_Ngz\,\psi _n(z) = E_n\psi _n^{(0)}(z)\>. \end{aligned}$$

The characteristic length scale

77 $$\begin{aligned} z_0 = \root 3 \of {\frac{\hbar ^2}{2m_N^2g}} = 5.87\,\mu \text {m}\>, \end{aligned}$$

and energy scale $E_0$ = $\root 3 \of {\hbar ^2 m_Ng^2/2}$ are given by the mass $m_N$ of the neutron and the acceleration of the Earth *g*. With the substitution

78 $$\begin{aligned} \sigma = \root 3 \of {\frac{2m_N^2g}{\hbar ^2}}\left( z - \frac{E_n}{m_Ng}\right) \equiv \frac{z - z_n}{z_0}\>, \end{aligned}$$

Eq. (76) becomes Airy’s equation

79 $$\begin{aligned} \frac{d^2{{\tilde{\psi }}}_n(\sigma )}{d\sigma ^2} - \sigma \,{{\tilde{\psi }}}_n(\sigma ) = 0\>. \end{aligned}$$

From the effective dilaton potential

80 $$\begin{aligned} V_\text {eff}(\phi ) = V_0\,e^{-\lambda \phi /m_\text {Pl}} + \frac{A_2\rho }{2m_\text {Pl}^2}\,\phi ^2\>, \end{aligned}$$

we can deduce the semi-classical neutron-dilaton coupling

81 $$\begin{aligned} V_\text {eff} = \frac{A_2}{2}\frac{m_N}{m_\text {Pl}^2}\,\psi ^*\psi \,\phi ^2\>. \end{aligned}$$

There are some subtleties involved here of a nature similar as in the case of the symmetron. We refer to [24] for further information. The corresponding quantum mechanical perturbation potential is given by

82 $$\begin{aligned} \text {V} = \frac{A_2}{2}\frac{m_N}{m_\text {Pl}^2}\,\phi ^2\>, \end{aligned}$$

and leads to a resonance frequency shift to first order (see e.g. [35]):

83 $$\begin{aligned} \delta E_{mn}^{(1)}&\equiv E_m^{(1)} - E_n^{(1)} \nonumber \\&= \frac{A_2}{2}\frac{m_N}{m_\text {Pl}^2}\int _{-\infty }^\infty dz\,\Big (\big |\psi _m^{(0)}(z)\big |^2 - \big |\psi _n^{(0)}(z)\big |^2\Big )\,\phi ^2(z)\>. \end{aligned}$$

Likewise, the first order correction to the wavefunctions reads (see e.g. [35])

84 $$\begin{aligned} \Psi _n^{(1)}({{\textbf {x}}},t) = \sum _{m\ne n}\frac{\displaystyle \int d^3x'\,\psi _m^{(0)*}({{\textbf {x}}}')\text {V}\psi _n^{(0)}({{\textbf {x}}}')}{E_n^{(0)} - E_m^{(0)}}\,\psi _m^{(0)}({{\textbf {x}}})\,e^{-\frac{i}{\hbar }E_n t}\>. \end{aligned}$$

Hence, the correction to the density $\displaystyle \varrho _n^{(0)}(z)=\psi _n^{(0)*}(z)\psi _n^{(0)}(z)$ to first order is given by

85 $$\begin{aligned} \varrho _n^{(1)}(z)&= 2\,\mathfrak {Re}\big (\psi _n^{(0)*}(z)\psi _n^{(1)}(z)\big ) \nonumber \\&= A_2\,\frac{m_N}{m_\text {Pl}^2}\,\mathfrak {Re}\sum _{m\ne n}\frac{\displaystyle \int _{-\infty }^\infty dz'\,\psi _m^{(0)*}(z')\,\psi _n^{(0)}(z')\,\phi (z')^2}{E_n^{(0)} - E_m^{(0)}} \nonumber \\&\quad \times \psi _n^{(0)*}(z)\psi _m^{(0)}(z)\>, \end{aligned}$$

where $\mathfrak {Re}$ denotes the real part.

In the one mirror case the unperturbed normalized wavefunction for $z>0$ reads (see e.g. [36])

86 $$\begin{aligned} \psi _n^{(0)}(z) = \frac{\text {Ai}\Big (\displaystyle \frac{z - z_n}{z_0}\Big )}{\sqrt{z_0}\,\text {Ai}'\Big (-\displaystyle \frac{z_n}{z_0}\Big )} \end{aligned}$$

with $z_n = E_n/(mg)$. Outside this region the wavefunction vanishes. For a single mirror filling the region $z \le 0$ we can use Eq. (34) and obtain for the resonance frequency shift

87 $$\begin{aligned} \delta E_{mn}^{(1)}&= \frac{A_2}{2}\frac{m_N}{m_\text {Pl}^2}\frac{1}{z_0}\int _0^\infty dz\,\Big (\phi _V + \left( \phi _0 - \phi _V\right) e^{-\mu _Vz}\Big )^2 \nonumber \\&\quad \times \left\{ \frac{\displaystyle \text {Ai}\Big (\frac{z - z_m}{z_0}\Big )^2}{\text {Ai}'\Big (-\displaystyle \frac{z_m}{z_0}\Big )^2} - \frac{\displaystyle \text {Ai}\Big (\frac{z - z_n}{z_0}\Big )^2}{\text {Ai}'\Big (-\displaystyle \frac{z_n}{z_0}\Big )^2}\right\} \>. \end{aligned}$$

It is straightforward to find all the corresponding expressions in the two mirror case. These expressions are very elaborate in their full detail and hence we will refrain from reproducing them herein.

In order to account for the “screening” of the neutron itself for the extraction of the experimental limits, the transition energies should be replaced as follows

88 $$\begin{aligned} \delta E_{pq} \rightarrow {\mathfrak {Q}}\,\delta E_{pq}\>, \end{aligned}$$

where ${\mathfrak {Q}}$ is given in Eq. (69). The whole parameter space of dilatons can be efficiently constrained using the results obtained herein. The corresponding analysis has been carried out in parallel to this work [10], where the solutions obtained here for a one mirror setup are used to exclude regions of the dilaton parameter space.

Nevertheless, in Table 3 we summarize the resonance frequency shift for a range of dilaton parameters near the experimental sensitivity in the case of a single mirror.

**Table 3:** $\delta E_{41}^{(1)}$ for 1 mirror, viz. Eq. (87), for the value $\rho = 2.51$ g/cm$^3$

$\delta E_{14}^{(1)}$ [eV]	$A_2$	$\lambda$
$3.03121\times 10^{-21}$	$10^{35}$	$10^{25}$
$5.71509\times 10^{-16}$	$10^{35}$	$10^{30}$
$5.75507\times 10^{-21}$	$10^{35}$	$10^{35}$
$2.60699\times 10^{-21}$	$10^{40}$	$10^{25}$
$6.76315\times 10^{-12}$	$10^{40}$	$10^{30}$
$5.59563\times 10^{-16}$	$10^{40}$	$10^{35}$
$1.51542\times 10^{-24}$	$10^{45}$	$10^{25}$
$1.51550\times 10^{-14}$	$10^{45}$	$10^{30}$
$3.02715\times 10^{-13}$	$10^{45}$	$10^{35}$

As can be seen in this table, the deviation can be larger than the experimental uncertainty of order $3\times 10^{-15}$ eV for cases where for instance $A_2=10^{40}$ and $\lambda =10^{30}$ corresponding to $M\simeq 10^{-2}$ GeV and $\Lambda \simeq 10^{-3}$ eV, i.e. the dark energy scale. As a result the forthcoming results in [10] will test an interesting range of scales for both particle physics and cosmology.

## Dilaton-induced pressure in CASIMIR experiments

Here, we consider limits that can be obtained by the Casimir And Non-Newtonian force EXperiment (Cannex) [13] (see also [37–39]). This experiment consists of two parallel plates in a vacuum chamber and has been devised to measure the Casimir force and hypothetical fifth forces. A dilaton field would induce a pressure between those plates, which can be measured with high precision.

We approximate the setup in one dimension along the *z*-axis as follows. Between the upper surface of the fixed lower mirror at $z = 0$ and the lower surface of the movable upper mirror at $z = d$ there is vacuum. Then follows the upper mirror with thickness *D* and above that at $z > d + D$ there is vacuum again. In order to obtain the induced pressure for the Cannex setup, we can apply the force induced on a point particle by dilatons in Eq. (71). We obtain

89 $$\begin{aligned} \textbf{f}_\phi = -\rho _M\int _{-\infty }^\infty dx\int _{-\infty }^\infty dy\int _d^{d + D} dz\,\partial _z\ln A(\phi )\,\textbf{e}_z\>. \end{aligned}$$

Consequently, the pressure in *z*-direction is given by

90 $$\begin{aligned} P_z = -\rho _M\int _d^{d + D} dz\,\partial _z\ln A(\phi )\>. \end{aligned}$$

The corresponding integral is a surface term and hence trivially carried out with the final result

91 $$\begin{aligned} P_z = \rho _M\,\Big (\ln A\big (\phi (d)\big ) - \ln A\big (\phi (d + D)\big )\Big )\>. \end{aligned}$$

This agrees with (see e.g. [25])

92 $$\begin{aligned} \nabla ^\mu T_{\mu \nu } = \partial _\nu \ln A\,T\>, \end{aligned}$$

where $T=g^{\mu \nu }T_{\mu \nu }$ and which reduces for a static field configuration of $\phi$ to

93 $$\begin{aligned} \partial ^z T_{zz} = -\partial _z P_z = \partial _z\ln A\,\rho _M\>. \end{aligned}$$

In all models of consideration $A(\phi ) = 1 + \delta A(\phi )$ with $\delta A(\phi )\ll 1$, such that $\ln A(\phi ) \simeq \delta A(\phi )$. For dilatons Eq. (3) holds and we finally obtain the dilaton-induced pressure

94 $$\begin{aligned} P_z = \rho _M\,\frac{A_2}{2m_\text {Pl}^2}\left( \phi ^2(d) - \phi ^2(d + D)\right) . \end{aligned}$$

For $\phi (d)$ we employ the value $\phi _d$ at the mirror surface of the corresponding two mirror solution given in Eq. (50), while for $\phi (d + D)$ we can use the value $\phi _0$ at the mirror surface of the one mirror solution given in Eq. (36) instead. Finally, we obtain for the pressure

95 $$\begin{aligned} P_z = \rho _M\,\frac{A_2}{2m_\text {Pl}^2}\left\{ \phi _d^2 - \left( \frac{\mu _V\,\phi _V + \mu _M\,\phi _M}{\mu _V + \mu _M}\right) ^2\right\} . \end{aligned}$$

This results can be used to carry out a numerical analysis [10] where the exclusion regions of the dilaton parameter space are obtained from the Cannex experiment as we expect a sensitivity of $\vert P_z\vert \le 1$ nPa.

## Dilaton constraints by lunar laser ranging

In this section we analyze bounds on dilatons due to Lunar Laser Ranging (LLR), which provides three separate constraints [14] due to

1. the Nordvedt effect, which relates to a difference between the free fall acceleration of the Earth and Moon towards the Sun (equivalence principle violations),
2. deviations from the inverse-square law at distances comparable to the Earth–Moon separation and
3. time-variation of *G*.

Since the dilaton interaction is time-independent, it cannot induce a time-variation of the effective coupling *G*. Hence, only the first two constraints can lead to dilaton bounds and will be analyzed here.

### Constraints due to the Nordvedt effect

For the first LLR test we consider the acceleration of the Earth ( ) and Moon ( ) towards the Sun ( ). For the Earth we find 7496where $\textbf{r}_{AU}$ is the vector pointing from Sun to Earth, $\textbf{a}_G$ is the acceleration due to the Sun’s gravitational field and  the acceleration due to the dilaton field of the Sun and given by Eq. ( 74), which holds for a test particle in an outer field without screening. The screening of the Earth is taken into account by the second charge factor . Likewise, the acceleration of the Moon is given by97where $\textbf{r}_{AU}$ is again the vector pointing from Sun to Earth, which is approximately the vector pointing from Sun to Moon. Then, $\textbf{a}_G$ is the acceleration due to the Sun’s gravitational field and taken equal for Earth and Moon, such that any difference in acceleration between Earth and Moon, *viz.* an equivalence principle violation, is attributed to the dilaton field. For the equivalence principle violation quantified by the Eötvos parameter98we find in the case of dilatons99 Typically this is constrained at the $\eta _{\textrm{em}}\le 2\times 10^{-13}$ level by the LLR experiment [40].

### Constraints due to deviations from the inverse-square law

For the second LLR test we consider the precession of the lunar perigee caused by fifth forces. The corresponding Eq. (124) is derived in Appendix A and reads100It is derived in Appendix A. Here, $\delta f(R)$ is the acceleration caused by dilatons at the maximum Earth-Moon separation *R*, and given by 74101Using Eq. (101) in Eq. (100) leads to the central relation102to be compared with experimental results of order $6\times 10^{-12}$. The results of the numerical analysis for both tests are reproduced in the accompanying paper [10]. Typically the range of excluded values for $A_2$ and $\lambda$ will be such that a region of parameter where $A_2\simeq 10^{10}$ will be excluded. More details will be given in [10].

## Conclusion

We have derived approximate analytical solutions to the dilaton field theory in the presence of a one or two mirror system as well as for a sphere. The 1-dimensional equations of motion have been integrated in each case. The analytical results obtained herein provide the necessary input for the numerical study carried out in parallel to this work [10]. The latter provides bounds on dilatons by using results from three “experiments”. First, from *q*BOUNCE we obtain results by employing bouncing ultracold neutrons, second, Cannex provides bounds by measuring induced pressures between parallel plates and third, from Lunar Laser Ranging we obtain additional bounds in the astrophysical regime. We have shown here that the dilaton models will be mostly tested in the region of parameter space where $\lambda \sim \sqrt{A_2}$. The constant $\lambda$ determines the steepness of the dilaton runaway potential whilst $A_2$ is the quadratic coupling constant to matter. Moreover, the different experiment will test different typical values of the parameter space. Solar system experiments such as Lunar Laser Ranging measuring the violation of the equivalence principle in the Sun–Earth–Moon system is mostly sensitive to values of $A_2\sim 10^{10}$ corresponding to masses of the dilaton in the solar system around $10^{-23}$ eV, i.e. with an interaction range of the order of 100 astronomical units. Similarly laboratory experiments are sensitive to values of $\lambda$ of the order of $10^{30}$ corresponding to a mass which is typically around $10^{-3}$ eV and a sub-millimeter range. The fact that the tests of the dilaton select an interaction range adapted to the typical size of the experiment is already what happens in the symmetron case [27]. Finally, all the dilaton models which can be tested experimentally satisfy the swampland conjectures [28] and therefore qualify as potential effective field theories which could have a fundamental origin.

## Data Availability

This manuscript has no associated data or the data will not be deposited. [Authors’ comment: The data that support the plots within this paper and results from calculations can be requested from the corresponding author.]

## Appendix A: Precession of the lunar perigee induced by fifth forces

In this Appendix we derive the precession of the lunar perigee induced by an arbitrary “fifth force”. Historically, the following result is essentially due to *Isaac Newton* ([41], Propositions XLIII–XLV). For a modern derivation see [42]. In this Appendix, any “force” is understood as the absolute value of the centripetal force per mass

103 $$\begin{aligned} f(r) = -\frac{\textbf{r}}{r}\cdot \frac{d\textbf{v}}{dt}\>. \end{aligned}$$

We recall the Binet equation governing $u = 1/r = g(\varphi )$ (see e.g. [42])

104 $$\begin{aligned} f(r)&= h^2u^2\frac{d^2u}{d\varphi ^2} + h^2u^3 \nonumber \\&= h^2g^2(\varphi )\frac{d^2g}{d\varphi ^2}(\varphi ) + h^2g^3(\varphi )\>, \end{aligned}$$

where *h* is the absolute value of $\textbf{h} = \textbf{r} \times \textbf{v}$.

First, we address the following question. Given that $u = 1/r = g(\varphi )$ is the inverse polar equation of an orbit described under the action of a centripetal force *f*(*r*) with a constant of areas *h*, what is the centripetal force ${{\tilde{f}}}(r)$ under which a revolving orbit $u = 1/r = g({{\tilde{\varphi }}}/\alpha )$ with ${{\tilde{\varphi }}} = \alpha \varphi$ and a constant of areas ${{\tilde{h}}} = \alpha h$ would be described, where $\alpha$ is some assigned constant? The polar form of an ellipse with its center at one focus and $\varphi$ defined such that $\varphi = 0$ corresponds to the point nearest to this focus, is given by

105 $$\begin{aligned} \frac{p}{r} = 1 + e\cos \varphi \end{aligned}$$

with eccentricity *e* and semi-latus rectum *p*. Hence, we have for an ellipse

106 $$\begin{aligned} f(r) = \frac{h^2}{p}\frac{1}{r^2}\>. \end{aligned}$$

We now require the centripetal force ${{\tilde{f}}}(r)$ under which the orbit

107 $$\begin{aligned} r = g({{\tilde{\varphi }}}/\alpha ) \end{aligned}$$

will be described with a constant of areas

108 $$\begin{aligned} {{\tilde{h}}} = \alpha h\>. \end{aligned}$$

We find

109 $$\begin{aligned} {{\tilde{f}}}(r)&= {{\tilde{h}}}^2u^2\frac{d^2u}{d{{\tilde{\varphi }}}^2} + {{\tilde{h}}}^2u^3 \nonumber \\&={{\tilde{h}}}^2g^2({{\tilde{\varphi }}}/\alpha )\frac{d^2g}{d{{\tilde{\varphi }}}^2}({{\tilde{\varphi }}}/\alpha ) +{{\tilde{h}}}^2g^3({{\tilde{\varphi }}}/\alpha ) \nonumber \\&= \alpha ^2 h^2g^2(\varphi )\frac{1}{\alpha ^2}\frac{d^2g}{d\varphi ^2}(\varphi ) + \alpha ^2 h^2g^3(\varphi ) \nonumber \\&= h^2g^2(\varphi )\frac{d^2g}{d\varphi ^2}(\varphi ) + \alpha ^2 h^2g^3(\varphi ) \nonumber \\&= f(r) - h^2g^3(\varphi ) + \alpha ^2 h^2g^3(\varphi ) \nonumber \\&= f(r) - h^2u^3 + \alpha ^2 h^2u^3\>, \end{aligned}$$

and finally *Newton’s theorem of revolving orbits* [43]

110 $$\begin{aligned} {{\tilde{f}}}(r) - f(r) = \frac{{{\tilde{h}}}^2 - h^2}{r^3}\>. \end{aligned}$$

In order to relate fifth forces to the precession of the Lunar perigee, we compare the elliptic orbit described under an inverse-square law of attraction with a nearly circular orbit caused by an inverse-square law of attraction together with a small fifth force. The elliptic orbit described by Eq. (105) is not revolving, while an additional fifth force leads to a deformation of the orbit and also lets the orbit revolve. Actually, the orbit of the Moon is to first order an ellipse with manifold corrections at higher orders. These higher orders also lead to corrections of the relation between fifth force and precession of the Lunar perigee, which are sub-leading and hence will be neglected.

For an elliptic orbit we have Eq. (106) and find for the force causing the revolving orbit

111 $$\begin{aligned} {{\tilde{f}}}(r)&= \frac{h^2}{p}\frac{1}{r^2} + \frac{{{\tilde{h}}}^2 - h^2}{r^3} \nonumber \\&= \frac{1}{p}\frac{1}{r^3}\left[ rh^2 + p\left( {{\tilde{h}}}^2 - h^2\right) \right] . \end{aligned}$$

For a nearly circular orbit, we may write

112 $$\begin{aligned} r = R - \delta r\>, \end{aligned}$$

where *R* is the maximum distance, which for an ellipse is $R = a(1 + e)$. Hence, we find

113 $$\begin{aligned} {{\tilde{f}}}(r) = \frac{1}{p}\frac{1}{r^3}\left[ \left( R - \delta r\right) h^2 + p\left( {{\tilde{h}}}^2 - h^2\right) \right] . \end{aligned}$$

The new force we describe in the form

114 $$\begin{aligned} {{\tilde{f}}}(r) = \frac{1}{p}\frac{C(r)}{r^3}\>, \end{aligned}$$

where *C*(*r*) is an arbitrary function. Since we consider nearly circular orbits, we perform a Taylor expansion

115 $$\begin{aligned} {{\tilde{f}}}(r) \simeq \frac{1}{p}\frac{C(R) - C'(R)\delta r}{r^3}\>, \end{aligned}$$

where $\displaystyle C'(R) = \frac{dC}{dR}$. Eq. (113) leads to

116 $$\begin{aligned} \left( R - \delta r\right) h^2 + p\left( {{\tilde{h}}}^2 - h^2\right) = C(R) - C'(R)\delta r\>. \end{aligned}$$

Comparing coefficients gives

117 $$\begin{aligned} Rh^2 + p\left( {{\tilde{h}}}^2 - h^2\right)&= C(R)\>, \nonumber \\ h^2&= C'(R)\>. \end{aligned}$$

For nearly circular orbits we have to leading order $p = R$ and hence

118 $$\begin{aligned} R{{\tilde{h}}}^2&= C(R)\>, \nonumber \\ h^2&= C'(R)\>. \end{aligned}$$

For $\alpha$ we find

119 $$\begin{aligned} \alpha = \frac{{{\tilde{h}}}}{h} = \sqrt{\frac{1}{R}\frac{C(R)}{C'(R)}}\>. \end{aligned}$$

Using Eq. (114) we can express $\alpha$ in terms of ${{\tilde{f}}}(r)$

120 $$\begin{aligned} \alpha = \sqrt{\frac{{{\tilde{f}}}(R)}{3{{\tilde{f}}}(R) + R{{\tilde{f}}}'(R)}}\>. \end{aligned}$$

Next, we consider the case of gravitational attraction due to the Earth and an additional small fifth force $\delta f(r)$ acting in radial direction and caused by the Earth. Hence, we have

121

To leading order we obtain

122

The correction to the precession of the Lunar perigee is obviously given by

123 $$\begin{aligned} \frac{\delta \Omega }{\Omega } = \alpha - 1\>, \end{aligned}$$

so we can finally connect the fifth force with the precession of the Lunar perigee

124

## References

[1] Joyce A, Jain B, Khoury J, Trodden M (2015). Beyond the cosmological standard model. Phys. Rep..

[2] Khoury J, Weltman A (2004). Chameleon cosmology. Phys. Rev. D.

[3] Khoury J, Weltman A (2004). Chameleon fields: awaiting surprises for tests of gravity in space. Phys. Rev. Lett..

[4] Brax P, van de Bruck C, Davis A-C, Khoury J, Weltman A (2004). Detecting dark energy in orbit: the cosmological chameleon. Phys. Rev. D.

[5] Damour T, Polyakov AM (1994). The string dilaton and a least coupling principle. Nucl. Phys. B.

[6] Babichev E, Deffayet C, Ziour R (2009). k-Mouflage gravity. Int. J. Mod. Phys. D.

[7] Brax P, Burrage C, Davis A-C (2013). Screening fifth forces in k-essence and DBI models. JCAP.

[8] Brax P, Valageas P (2014). K-mouflage cosmology: the background evolution. Phys. Rev. D.

[9] Vainshtein AI (1972). To the problem of nonvanishing gravitation mass. Phys. Lett..

[10] *to be published*

[11] Abele H, Jenke T, Leeb H, Schmiedmayer J (2010). Ramsey’s method of separated oscillating fields and its application to gravitationally induced quantum phaseshifts. Phys. Rev. D.

[12] Jenke T, Geltenbort P, Lemmel H, Abele H (2011). Realization of a gravity-resonance-spectroscopy technique. Nat. Phys..

[13] Sedmik R, Brax P (2018). Status report and first light from cannex: casimir force measurements between flat parallel plates. J. Phys. Conf. Ser..

[14] K. Nordtvedt, Lunar laser ranging—a comprehensive probe of the post-newtonian long range interaction, in *Gyros, Clocks, Interferometers...: Testing Relativistic Graviy in Space*, ed. by C. Lämmerzahl, C.W. Francis Everitt, F.W. Hehl (Springer, Berlin, 2001), pp. 317–329

[15] Brax P, van de Bruck C, Davis A-C, Shaw D (2010). The dilaton and modified gravity. Phys. Rev. D.

[16] Brax P, van de Bruck C, Davis A-C, Li B, Shaw DJ (2011). Nonlinear structure formation with the environmentally dependent dilaton. Phys. Rev. D.

[17] Kraiselburd L, Landau SJ, Salgado M, Sudarsky D, Vucetich H (2018). Equivalence principle in chameleon models. Phys. Rev. D.

[18] Hinterbichler K, Khoury J, Levy A, Matas A (2011). Symmetron cosmology. Phys. Rev. D.

[19] Gasperini M, Piazza F, Veneziano G (2002). Quintessence as a runaway dilaton. Phys. Rev. D.

[20] Damour T, Piazza F, Veneziano G (2002). Violations of the equivalence principle in a dilaton runaway scenario. Phys. Rev. D.

[21] Damour T, Piazza F, Veneziano G (2002). Runaway dilaton and equivalence principle violations. Phys. Rev. Lett..

[22] Brax P, van de Bruck C, Davis A-C, Shaw D (2010). Dilaton and modified gravity. Phys. Rev. D.

[23] Ivanov AN, Cronenberg G, Höllwieser R, Pitschmann M, Jenke T, Wellenzohn M, Abele H (2016). Exact solution for chameleon field, self-coupled through the Ratra–Peebles potential with $$n=1$$ and confined between two parallel plates. Phys. Rev. D.

[24] Brax P, Pitschmann M (2018). Exact solutions to nonlinear symmetron theory: one- and two-mirror systems. Phys. Rev. D.

[25] Pitschmann M (2021). Exact solutions to nonlinear symmetron theory: one- and two-mirror systems. II. Phys. Rev. D.

[26] Brax P, Casas S, Desmond H, Elder B (2021). Testing screened modified gravity. Universe.

[27] Cronenberg G, Brax P, Filter H, Geltenbort P, Jenke T, Pignol G, Pitschmann M, Thalhammer M, Abele H (2018). Acoustic Rabi oscillations between gravitational quantum states and impact on symmetron dark energy. Nat. Phys..

[28] Ooguri H, Palti E, Shiu G, Vafa C (2019). Distance and de Sitter Conjectures on the Swampland. Phys. Lett. B.

[29] Brax P, van de Bruck C, Davis A-C (2020). Swampland and screened modified gravity. Phys. Rev. D.

[30] Brax P, Burrage C, Davis A-C (2018). Laboratory constraints. Int. J. Mod. Phys. D.

[31] Burrage C, Elder B, Millington P, Saadeh D, Thrussell B (2021). Fifth-force screening around extremely compact sources. J. Cosmol. Astropart. Phys..

[32] Jenke T (2014). Gravity resonance spectroscopy constrains dark energy and dark matter scenarios. Phys. Rev. Lett..

[33] Nesvizhevsky VV (2002). Quantum states of neutrons in the Earth’s gravitational field. Nature.

[34] Westphal A, Abele H, Baessler S, Nesvizhevsky VV, Petukhov AK, Protasov KV, Voronin AY (2007). A quantum mechanical description of the experiment on the observation of gravitationally bound states. Eur. Phys. J. C.

[35] Landau LD, Lifshitz EM (1991). Quantenmechanik. Lehrbuch der theoretischen Physik III.

[36] M. Pitschmann, H. Abele, Schrödinger equation for a non-relativistic particle in a gravitational field confined by two vibrating mirrors. **12** (2019)

[37] Klimchitskaya GL, Mostepanenko VM, Sedmik RIP, Abele H (2019). Prospects for searching thermal effects, non-Newtonian gravity and axion-like particles: cannex test of the quantum vacuum. Symmetry.

[38] Klimchitskaya GL, Mostepanenko VM, Sedmik RIP (2019). Casimir pressure between metallic plates out of thermal equilibrium: proposed test for the relaxation properties of free electrons. Phys. Rev. A.

[39] Sedmik RIP, Pitschmann M (2021). Next generation design and prospects for cannex. Universe.

[40] Williams JG, Turyshev SG, Boggs D (2012). Lunar laser ranging tests of the equivalence principle. Class. Quantum Gravity.

[41] Newton I (1999). The Principia: Mathematical Principles of Natural Philosophy.

[42] Chandrasekhar S (1995). Newton’s Principia for the Common Reader.

[43] Whittaker ET (1988). A Treatise on the Analytical Dynamics of Particles and Rigid Bodies.

